# Primary Effusion Lymphoma without an Effusion: A Rare Case of Solid Extracavitary Variant of Primary Effusion Lymphoma in an HIV-Positive Patient

**DOI:** 10.1155/2018/9368451

**Published:** 2018-01-28

**Authors:** Hamza Hashmi, Drew Murray, Samer Al-Quran, William Tse

**Affiliations:** ^1^Division of Hematology and Oncology, University of Louisville, Louisville, KY, USA; ^2^Department of Pathology and Laboratory Medicine, University of Louisville, Louisville, KY, USA; ^3^Division of Blood and Marrow Transplant, University of Louisville, Louisville, KY, USA

## Abstract

Primary effusion lymphoma (PEL) is a unique form of non-Hodgkin lymphoma, usually seen in severely immunocompromised, HIV-positive patients. PEL is related to human herpesvirus-8 (HHV-8) infection, and it usually presents as a lymphomatous body cavity effusion in the absence of a solid tumor mass. There have been very few case reports of HIV-positive patients with HHV-8-positive solid tissue lymphomas not associated with an effusion (a solid variant of PEL). In the absence of effusion, establishing an accurate diagnosis can be challenging, and a careful review of morphology, immunophenotype, and presence of HHV-8 is necessary to differentiate from other subtypes of non-Hodgkin lymphoma. Treatment involves intensive chemotherapy, and prognosis is usually poor. We present a rare case of a PEL variant in an HIV-positive patient who presented with extensive lymphadenopathy without any associated effusions.

## 1. Introduction

Primary effusion (PEL) is an uncommon subtype of non-Hodgkin lymphoma seen in patients with human immunodeficiency virus (HIV). It is associated with human herpesvirus type 8 (HHV-8) also known as Kaposi sarcoma herpesvirus (KSHV). There is a rare solid extracavitary variant that can be mistaken morphologically for other types of aggressive diffuse large B-cell lymphoma (DLBCL).

## 2. Case Presentation

A 51-year-old Caucasian male, homosexual with a past medical history significant for controlled hypertension and ulcerative colitis treated with infliximab, presented with fatigue, intermittent night sweats, and significant weight loss of about 70 lbs over a period of 3 months. The patient reported productive cough and was nonbloody during this period of time accompanied by dry itchy skin with easy bruising.

**Figure 1:**
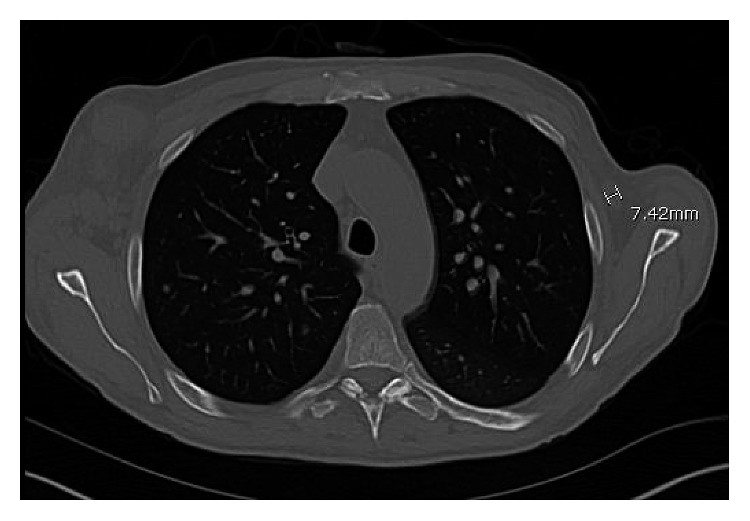
CT chest showing a 5.6 cm × 4.9 cm lymph node mass in the right axilla.

**Figure 2:**
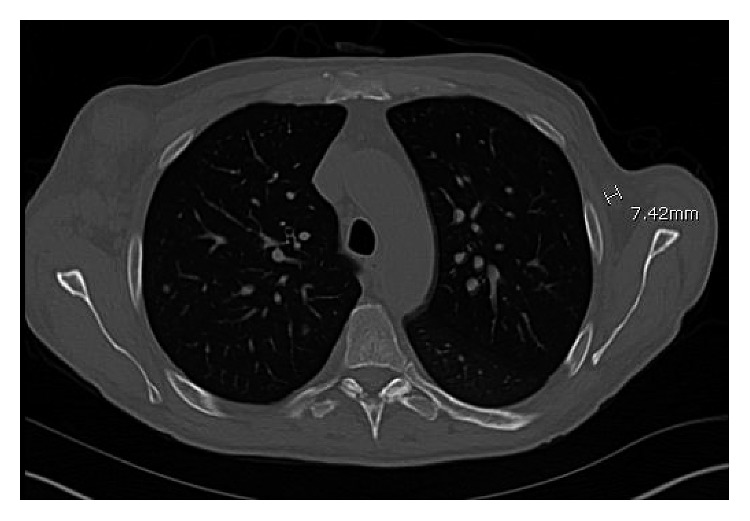
CT chest showing a 0.7 cm lymph node in the left axilla.

**Figure 3:**
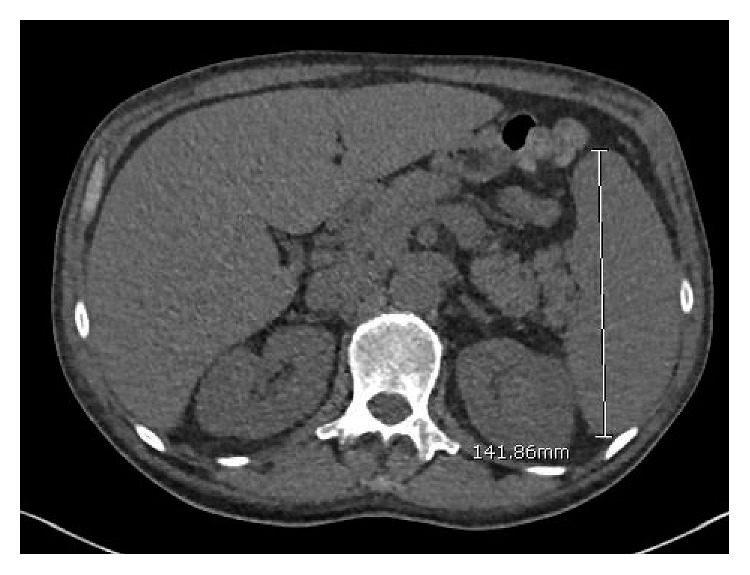
CT abdomen showing splenic enlargement of 14.8 cm.

CT scan of the chest, abdomen, and pelvis performed showed lymphadenopathy in the axillary area (Figures 1 and 2), lymphadenopathy in the abdominal cavity, and splenomegaly of 14.8 cm (Figure 3). Complete blood count revealed pancytopenia with white blood cell count of 9.8 × 10^3^/microL, hemoglobin of 7.4 g/dl, and platelet count of 65,000 × 10^3^/microL. INR was normal. Serum protein electrophoresis and immunofixation were normal. Serum HIV testing was positive with a high level of viral load, 5,900,000 copies/ml, CD4 count of 14 cells/mm^3^, and CD8 count of 78 cells/mm^3^. Serum Epstein–Barr virus testing was positive with a viral load of 14,000 copies/ml. Serum HHV-8 testing was positive with a viral load of 2,400,000 copies/ml. Whole-body PET scan revealed index lesions in the right axillary lymph node of 5.8 × 4.5 cm with a maximal SUV of 19.98 and a 6.6 × 5.5 cm left perisplenic mass with an SUV of 15.01. Left inguinal lymph node and right supraclavicular lymph node areas also showed mild PET avidity. Infectious disease service was consulted, and the patient was started on antiretroviral therapy with abacavir/lamivudine/dolutegravir. Given his extensive lymphadenopathy, the patient underwent a bone marrow biopsy as well as axillary lymph node biopsy.

**Figure 4:**
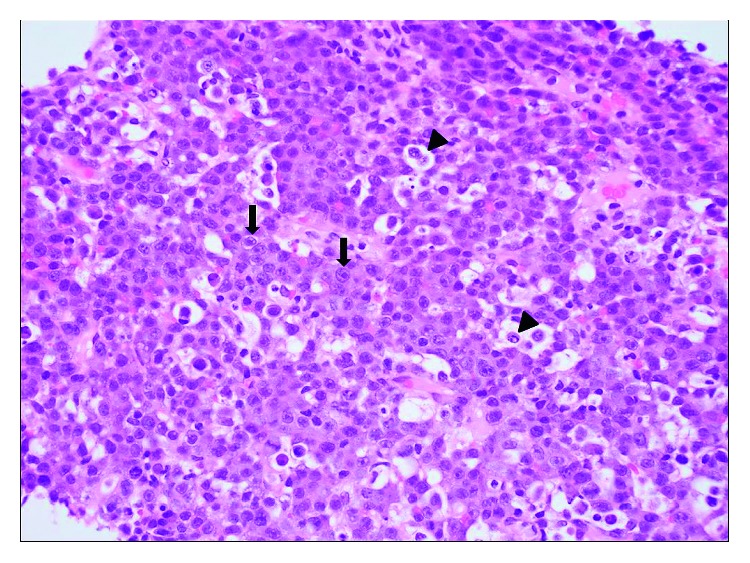
High-power photomicrograph of a hematoxylin and eosin- (H/E-) stained histologic section showing effacement of the lymph node architecture by the proliferation of immunoblastic cells (arrows) with abundant amphophilic cytoplasm, eccentric round large nuclei, and prominent single macronucleoli. Scattered apoptotic bodies and tangible body macrophages (arrowheads) were seen in background (original magnification: ×400).

**Figure 5:**
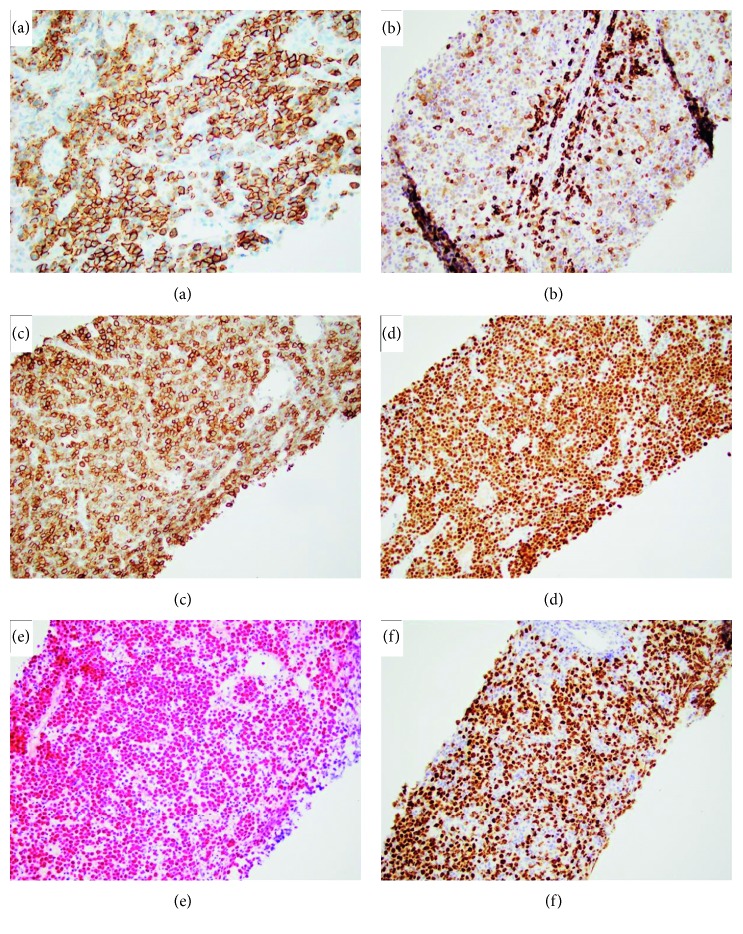
Composite photomicrograph of immunohistochemical and in situ hybridization studies showing that tumor cells are positive for (a) CD138 (variable), (b) CD79a (weak, variable), (c) CD30, (d) MUM1, (e) EBV (EBER), and (f) HHV-8.

A needle core of an axillary lymph node was performed. Microscopic examination of the histologic section showed diffuse effacement of the lymph node tissue architecture by the proliferation of immunoblastic cells with abundant amphophilic cytoplasm, eccentric round large nuclei, and prominent single macronucleoli (Figure 4). There was variable nuclear pleomorphism, with brisk mitotic activity and scattered apoptotic bodies and tangible body macrophages. Immunohistochemical studies (Figure 5) were performed with appropriate positive and negative controls according to established protocols. They demonstrated that the neoplastic cells were strongly CD30 (+), MUM1 (+), and diffusely HHV-8 (+) (using anti-LANA (latency-associated nuclear antigen) antibody), with high labeling of nuclei by Ki-67 (>95%), variable expression of CD138, and focal expression of CD45, CD79a, and p53. In situ hybridization study for EBV-encoded RNA (EBER) was diffusely positive. The neoplastic cells were negative for PAX5, CD117, CD56, ALK1, cyclin-D1, BCL2, BCL6, CD3, CD5, cytokeratin AE1/AE3, p63, CD20, S100 protein, CD15, and CD10. In situ hybridization studies for kappa and lambda light chain mRNA were negative. A portion of the fresh biopsy tissue was submitted for flow cytometric analysis which demonstrated the presence of large cells with bright expression of CD38. Cytogenetics performed revealed a normal male karyotype 46,XY.

The overall morphologic and immunophenotypic findings supported the diagnosis of an extracavitary (solid) variant of primary effusion lymphoma (PEL). The bone marrow biopsy did not reveal any evidence of lymphoma. There was no clinical, morphological, or radiographic evidence of other HIV-associated infectious or malignant complications including Kaposi sarcoma.

The patient was admitted to the hospital, and within 96 hours of starting his antiretroviral therapy, he received a first cycle of EPOCH (etoposide, prednisone, vincristine, cyclophosphamide, and doxorubicin). Two days after completing the first cycle, he developed neutropenic fever and acute hypoxic respiratory failure. He was started on broad-spectrum antibiotics and eventually required endotracheal intubation and mechanical ventilation. As CT scan of the chest revealed ground glass opacities, he was empirically started on Bactrim and prednisone. Bronchoscopy with bronchoalveolar lavage was performed which revealed PCR-positive pneumocystis. His clinical course was further complicated by septic shock by *Staphylococcus epidermidis* bacteremia, and the patient required vasopressor support along with broad-spectrum antibiotics. Antiretroviral therapy was continued throughout the course. Despite aggressive measures, his clinical condition continued to decline. As the patient experienced several episodes of generalized tonic-clonic seizures, CT head was done which showed right-sided intracranial bleed. Neurosurgery was consulted, and the patient was started on protamine drip for reversal of the possible subcutaneous heparin effect. Given large hemorrhagic stroke, significant comorbidities including aggressive lymphoma, and overall poor prognosis, his family decided to pursue comfort care, and the patient passed away shortly after extubation. Although the patient was started on antiretroviral therapy within 96 hours and administered chemotherapy within one week from his HIV and primary effusion lymphoma (solid variant) diagnosis, his disease had an aggressive clinical course leading to death within 2 weeks after completing the first cycle of chemotherapy.

## 3. Case Discussion

### 3.1. Epidemiology

An estimated 5–20% of patients with human immunodeficiency virus (HIV)/acquired immunodeficiency syndrome (AIDS) will develop an NHL over their lifetime [1]. PEL, the more common serous form of KSHV large B-cell lymphoma, constitutes 1–4% of all HIV/AIDS-associated NHLs [2]. Due to its rarity and late discovery of KSHV in 1994, less than 100 cases of a solid variant of PEL have been reported to date. Although exceedingly uncommon PEL and its solid variant have been identified in patients without HIV/AIDS, these patients are typically HHV-8 negative and present with an existing immunocompromised state such as advanced age, malignancy, cirrhosis, hepatitis, immunosuppressant medications, and existing malignancies [3].

### 3.2. Presentation and Diagnostic Evaluation

Among HIV-associated NHLs, a solid variant of PEL primarily presents in extracavitary sites such as the lymph nodes, gastrointestinal tract, pharyngeal lymphoid tissue, bone marrow, liver, central nervous system, and spleen without the development of lymphomatous effusions seen in PEL [4]. A 2005 study noted that 100% of patients with a solid variant of PEL presented with generalized lymphadenopathy while only occurring in 20% of those with PEL [4]. Abnormal laboratory findings include for anemia, elevated LDH levels, and hypoalbuminemia due to development of nephrotic syndrome [4].

After initial evaluation of renal function, hepatic function, blood counts, electrolytes, and serum lactate dehydrogenase levels, diagnosis is helped by blood viral testing for HIV and HHV-8. Imaging with CT of the chest, abdomen, and pelvis or PET scan is used for evaluation of the extent of the disease. Biopsy of suspicious lesions is required for diagnosis, through morphologic examination and immunophenotyping by flow cytometry or immunohistochemistry. Pleural effusions should be analyzed for HHV-8 as positive pleural fluid is a characteristic of PEL.

Extracavitary (solid variant) PEL presents with solid masses exhibiting similar morphology, immunophenotype, and gene expression profiles to classic PEL, which arises predominantly as a lymphomatous effusion within body cavities, and is characteristically associated with HHV-8 infection. The major differential diagnosis includes plasmablastic lymphoma, a variant of large B-cell lymphoma with an unfavorable outcome that is commonly associated with HIV/immune suppression. These two entities have overlapping morphologic and immunophenotypic features; however, the presence of HHV-8 infection supports the diagnosis of extracavitary PEL.

## 4. Prognosis

It is not entirely clear whether the prognosis is better with a solid variant of PEL as compared to the classic PEL. One comparative study of 8 patients with extracavitary PEL and 29 with classic PEL suggested a better prognosis for extracavitary PEL (median survival rate, 11 months versus 3 months) [5]. Another study showed median overall survival of 10.2 months which was not different between groups but showed higher disease-free survival in the extracavitary group [6].

Another study looking at patients treated with similar anthracycline-based therapy reported higher CR rates in patients with classic PEL (66%) versus extracavitary variant (40%). However, patients with classic PEL had higher rates of relapse (40%) at median follow-up of 25 months [6].

Based on a retrospective series of 28 HIV-infected patients with PEL, two prognostic factors have been identified that are independently associated with shorter survival: [2] the poor performance status and [3] the absence of preexisting HAART use [7].

## 5. Treatment

There is no standard of care established for the treatment of PEL, and prognosis remains dismal.

As in most patients with AIDS-associated lymphomas, the most common treatment used has been CHOP (cyclophosphamide, doxorubicin, vincristine, and prednisone) chemotherapy and concurrent HAART.

Initiation of HAART has been shown to be an essential component of successful therapeutic regimens in HIV+ patients with PEL. Retrospective case reports show prolonged survival and one case of complete remission in PEL treated with introduction of HAART [8, 9].

EPOCH (rituximab, etoposide, prednisone, vincristine, cyclophosphamide, and doxorubicin), with or without concomitant HAART, has also emerged as an alternative to CHOP and, in the context of HIV infection, might be associated with a slightly greater response rate [10].

Because PEL tumor cells rarely express CD20, rituximab has traditionally not been included in the treatment algorithms. If positive, rituximab can be employed as part of the treatment regimen.

The impact of combination antiretroviral therapy employed in patients with PEL has been documented [11]. However, awareness of the evolving knowledge of drug interactions with HAART, in particular protease inhibitors and chemotherapy, is important when creating a treatment plan [12].

Regardless of the treatment regimen, this immunocompromised population is at risk of opportunistic infections. Therefore, it is essential that they receive prophylactic antibiotics and, when the risk of febrile neutropenia exceeds 20%, adjuvant granulocyte colony-stimulating factor. Regardless of CD4 count, Bactrim should be used for PCP prophylaxis. In case of cytopenias related to chemotherapy or lymphoma, Bactrim can be substituted by pentamidine. Fluoroquinolone prophylaxis should be used in case of severe prolonged neutropenia [13, 14].

The potential efficacy of radiation therapy has been supported by a report of its successful use in a patient with chemotherapy-refractory PEL with solid pleural and chest masses who remained disease free at 12 months of follow-up. Hence, radiation therapy could be used in patients who are either refractory or relapsed to conventional chemotherapy [15].

More intensive regimens, such as high-dose chemotherapy followed by autologous hematopoietic cell transplantation (HCT) or allogeneic HCT, have had mixed results with two case reports showing success and another showing no response [16, 17]. The development of novel therapeutic agents for treatment of solid-variant PEL has not been reported in literature.
